# Draft Genome Sequence of an Endophytic *Actinoplanes* Species, Encoding Uncommon *trans*-Acyltransferase Polyketide Synthases

**DOI:** 10.1128/genomeA.00164-16

**Published:** 2016-03-24

**Authors:** Sara Centeno-Leija, Pablo Vinuesa, Karol Rodríguez-Peña, Miriam Trenado-Uribe, Yair Cárdenas-Conejo, Hugo Serrano-Posada, Romina Rodríguez-Sanoja, Sergio Sánchez

**Affiliations:** a Departamento de Biología Molecular y Biotecnología, Instituto de Investigaciones Biomédicas, Universidad Nacional Autónoma de México, Ciudad de México, Mexico; b Catedráticos CONACyT, Laboratorio de Bioingeniería, Universidad de Colima, Coquimatlán, Colima, Mexico; c Programa de Ingeniería Genómica, Centro de Ciencias Genómicas, Universidad Nacional Autónoma de México, Cuernavaca, Morelos, Mexico

## Abstract

*Actinoplanes* is an endophytic actinobacterium isolated from the medicinal plant *Amphipterygium adstringens*. The strain draft genome sequence reveals a gene cluster involved in the biosynthesis of a hybrid *trans*-acyltransferase (AT) polyketide, an unconventional bioactive metabolite never reported before in the genus *Actinoplanes*.

## GENOME ANNOUNCEMENT

Endophytic microorganisms from medicinal plants are recognized as a source of novel bioactive natural products (1, 2). *Actinoplanes* sp. was isolated from *Amphipterygium adstringens* (K. Rodríguez-Peña, M. Macías-Rubalcava, L. Rocha Zavaleta, R. Rodríguez-Sanoja, S. Sanchez, unpublished data), a medicinal plant that exhibits a broad range of biological properties, including antitumor, antimicrobial, and anti-inflammatory activities (3–5). Furthermore, *Actinoplanes* spp. are a group of actinobacteria known for producing pharmaceutically important compounds, such as the antimicrobial teicoplanin (6, 7) and anticancer (8) and antidiabetic drugs (9), emerging as an important genus to search for novel specialized metabolites by genome mining (9, 10).

The genome sequencing of *Actinoplanes* sp. was performed by the BaseClear Company (The Netherlands), using the Illumina HiSeq 2500 system. Approximately 508 Mb of raw data of 125 bp-long paired-end reads were generated, resulting in 54-fold coverage, with average quality scores (Phred) of 33.7. *De novo* assembly was performed using SPAdes version 3.5.0 (11). The resulting contigs were ordered with Ragout version 0.2 (12) using *Actinoplanes* sp. strain N902-109 (GenBank accession no. PRJNA198760) as the reference genome. The assembly was improved by remapping reads using Pilon version 1.12 (13). The draft genome sequence of 7,752,284 bp comprises 24 scaffolds, 7,161 protein-coding sequences, 67 RNAs, and a G+C content of 68.9%. Sequence analysis was performed by combining antiSMASH version 3.0.4 (14) and the PRISM genomic analysis platform (15). The analysis predicted 16 gene clusters coding for enzymes involved in the biosynthesis of secondary metabolites, including bacteriocins, type 1 and 3 polyketide synthases (PKS), and nonribosomal peptide synthases (NRPS). Remarkably, one gene cluster for the biosynthesis of an uncommon hybrid *trans*-acyltransferase (AT) polyketide was also found. *trans*-AT-PKS are an unconventional class of multimodular enzymes never reported before in *Actinoplanes*. These enzymes are frequently found in bacterial symbionts (16–18), which lack acyltransferase (AT) domains but instead receive its acyl precursor by a free-standing AT (18, 19). Sequence analysis performed using 2metDB, Pfam, and UniProt (20, 21) revealed a hybrid NRPS-t2PKS-*trans*-AT-PKS-t1PKS cluster, suggesting a highly complex biosynthetic pathway. The BLAST sequence analysis of the NRPS-t2PKS-*trans*-AT-PKS-t1PKS enzymes indicates a low level (<40%) of sequence identity compared to similar modules coded by the genomes of the *Paenibacillaceae* and *Peptococcaceae* families and the genera *Bacillus* and *Streptomyces*. These findings strongly support the presence of a novel hybrid *trans*-AT-PKS gene cluster. Since antitumor (22, 23) and antimicrobial activities (24) have been reported for *trans*-AT polyketides, the potential of this endophytic actinobacterium as a source of novel natural bioactive compounds is considerable.

### Nucleotide sequence accession numbers. 

The whole-genome shotgun (WGS) project of *Actinoplanes* sp. was deposited at DDBJ/EMBL/GenBank under the WGS project accession no. LOJP00000000. The version described in this paper is the first version, LOJP01000000.
